# The crystal structure of human ferroptosis suppressive protein 1 in complex with flavin adenine dinucleotide and nicotinamide adenine nucleotide

**DOI:** 10.1002/mco2.479

**Published:** 2024-02-26

**Authors:** Shijian Feng, Xiaofang Huang, Dan Tang, Xiaoyu Liu, Liang Ouyang, Dehua Yang, Kunjie Wang, Banghua Liao, Shiqian Qi

**Affiliations:** ^1^ Department of Urology and Institute of Urology (Laboratory of Reconstructive Urology) State Key Laboratory of Biotherapy and Cancer Center, West China Hospital, College of Life Sciences, Sichuan University Chengdu China; ^2^ The National Center for Drug Screening, Shanghai Institute of Materia Medica, Chinese Academy of Sciences Shanghai China

**Keywords:** 6‐OH‐FAD, ferroptosis, FSP1, NADH, oxidoreductase, structure

## Abstract

Ferroptosis is a recently discovered form of regulated cell death characterized by its distinct dependence on iron and the peroxidation of lipids within cellular membranes. Ferroptosis plays a crucial role in physiological and pathological situations and has attracted the attention of numerous scientists. Ferroptosis suppressive protein 1 (FSP1) is one of the main regulators that negatively regulates ferroptosis through the GPX4‐independent FSP1–CoQ10–NAD(P)H axis and is a potential therapeutic target for ferroptosis‐related diseases. However, the crystal structure of FSP1 has not been resolved, which hinders the development of therapeutic strategies targeting FSP1. To unravel this puzzle, we purified the human FSP1 (hFSP1) protein using the baculovirus eukaryotic cell expression system and solved its crystal structure at a resolution of 1.75 Å. Furthermore, we evaluated the oxidoreductase activity of hFSP1 with NADH as the substrate and identified E156 as the key amino acid in maintaining hFSP1 activity. Interestingly, our results indicated that hFSP1 exists and functions in a monomeric state. Mutagenesis analysis revealed the critical role of the C‐terminal domain in the binding of substrate. These findings significantly enhance our understanding of the functional mechanism of FSP1 and provide a precise model for further drug development.

## Graphical abstract

The structural section of hFSP1 is shown. The substrate clamp, NADH, and FAD are highlighted. Y296 and F360 comprise the substrate clamp of hFSP1. The clamp is highlighted using text. The NADH and FAD are shown in sticks and colored as green and magenta, respectively.

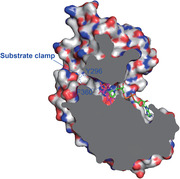

## 1 INTRODUCTION

Ferroptosis is a programmed cell death that differs from traditional forms of cell death, such as apoptosis and necrosis.^1^, ^2^ Unlike apoptosis, which involves controlled cell death without causing inflammation,^3^ or necrosis, which typically results from acute cellular injury and triggers an inflammatory response,^4^ ferroptosis is characterized by its dependence on iron and lipid peroxidation.^5^ This unique mode of cell death has attracted increasing attention due to its crucial role in both physiological and pathological conditions.^6^ For instance, it is involved in the regulation of tumor suppression, immune surveillance, and tissue injury/repair and regeneration.^7^, ^8^, ^9^, ^10^ Dysregulation of ferroptosis leads to tissue/organ dysfunction, inflammatory activation, and the occurrence and development of various diseases.^11^, ^12^, ^13^, ^14^ For instance, neurodegenerative disorders, ischemia/reperfusion injuries, and various types of cancers where cells are particularly vulnerable to lipid peroxidation‐induced damage have been reported to be closely linked to ferroptosis.^12^, ^15^, ^16^, ^17^, ^18^, ^19^, ^20^, ^21^, ^22^, ^23^, ^24^ Therefore, exploring the role and regulatory mechanisms of ferroptosis in these diseases is critical for novel therapeutic strategies and offers insights into the interplay between ferroptosis and cellular homeostasis.^24^

Ferroptosis is negatively regulated by glutathione peroxidase 4 (GPX4) and ferroptosis suppressor protein 1 (FSP1), also known as apoptosis‐inducing factor mitochondria‐associated 2 (AIFM2).^25^, ^26^ These proteins serve as critical guardians of cellular membrane integrity by counteracting lipid peroxidation.^27^ GPX4 is a selenoprotein that acts as an antioxidant enzyme, catalyzing the reduction of lipid hydroperoxides to alcohols, thereby preventing the buildup of toxic lipid peroxides in cell membranes.^28^ FSP1 was identified as a ferroptosis regulator in 2019 and has been emerging as a novel and intriguing component of ferroptosis signaling.^26^, ^27^ Unlike GPX4, which primarily functions in the cytoplasm and mitochondria, FSP1 localizes to mitochondria‐associated membranes (MAMs)^29^ and exerts antiferroptotic effects as a coenzyme Q10 (CoQ10) oxidoreductase.^26^ FSP1 reduces CoQ10 to its antioxidant form, ubiquinol, which subsequently acts as a lipid peroxidation scavenger within the mitochondrial membrane.^26^, ^27^

FSP1 provides an alternative route to counteract lipid peroxidation that operates independently of GPX4,^30^ suggesting that FSP1 is necessary for ferroptosis inhibition once GPX4 is compromised.^29^ Notably, FSP1 localizes in MAMs, indicating that it might be involved in regulating the interface between mitochondria and the endoplasmic reticulum and thereby regulating ferroptosis.^27^, ^31^ Although FSP1 is a key regulator of ferroptosis and several pharmacological agents targeting FSP1 have been discovered,^24^ the crystal structure of FSP1 is still unclear. This has hindered the further exploitation of pharmacological agents that can modulate FSP1 activity specifically and effectively. The development of novel compounds that can enhance or inhibit FSP1‐mediated protection against ferroptosis is necessary for therapeutic intervention but requires further investigation.^32^, ^33^

In this study, we reported the crystal structure of the human FSP1 protein (hFSP1) at a resolution of 1.75 Å. Based on the crystal structure of hFSP1, we further measured hFSP1 enzyme kinetic parameters and identified critical amino acids that are essential to hFSP1 activity. Together, these results here shed light on the molecular mechanism of FSP1 oxidoreductase activity and provide a precise model for drug development.

## 2 RESULTS

### 2.1 Expression, purification, and structural analysis of FSP1 protein

**FIGURE 1:**
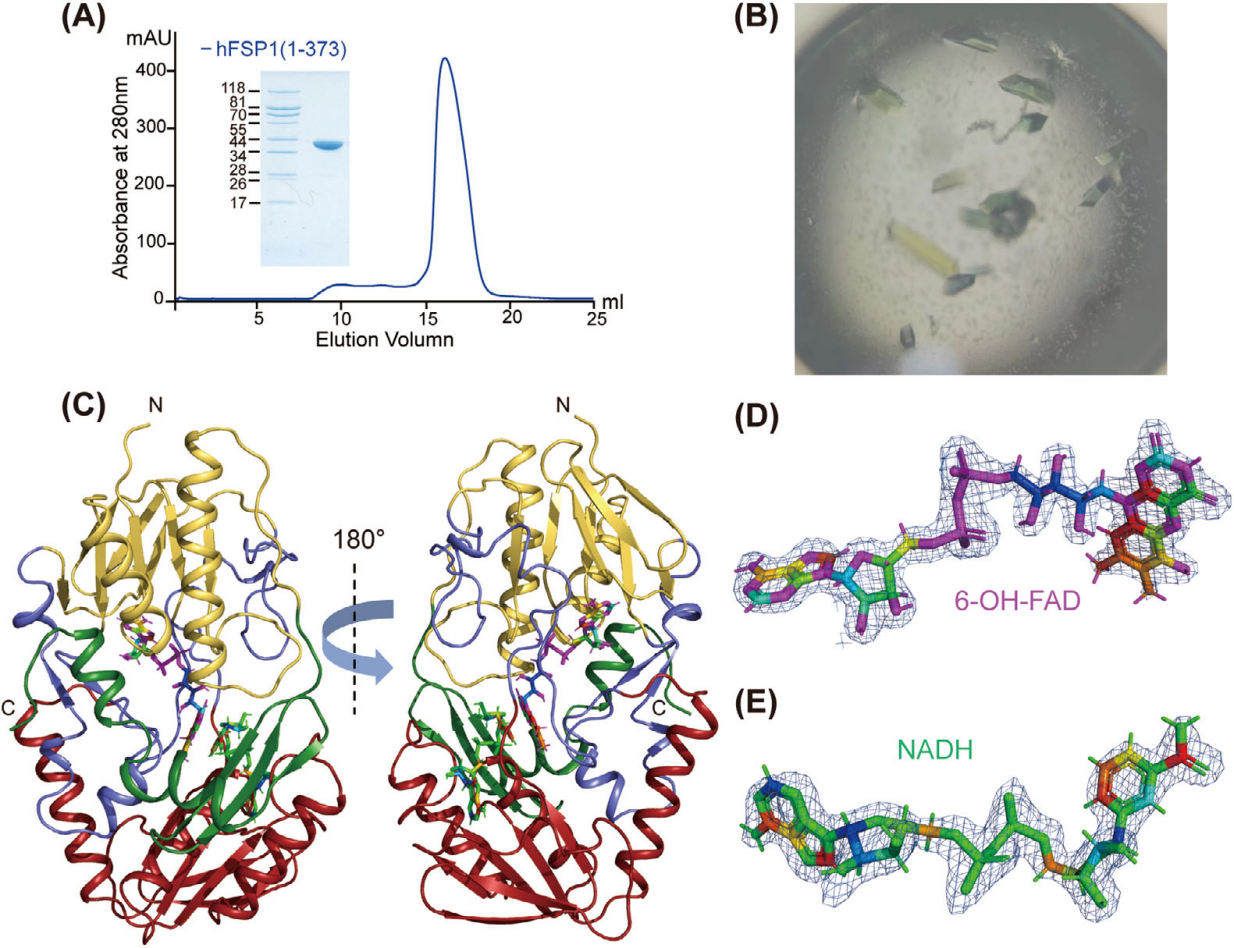
Crystal structure of human FSP1. (A) Gel filtration (Superdex 200 10/300 GL) profile of hFSP1. The horizontal axis is elution volume, and the vertical axis is ultraviolet (UV) absorption. The UV absorbance is shown as the blue line. The Coomassie blue‐stained sodium dodecyl sulfate‐polyacrylamide gel electrophoresis (SDS/PAGE) gel shows the peak fraction of hFSP1 from gel filtration. (B) Typical examples of crystals of hFSP1.hFSP1 crystals were rod‐shaped, and the estimated sizes ranged from ∼2 to 4  μm in the longest dimension. These images were taken using a Nikon optical microscope.(C) The overall structure of hFSP1 in complex with 6‐OH‐FAD and NADH. The structure is shown as a cartoon. Light blue, hFSP1; cyan, 6‐OH‐FAD; green, NADH. The N‐ and C‐termini of hFSP1 are labeled. (D and E) 2*F_O_*–*F_C_* electron density maps contoured at the 1.5 *σ* level approximately 6‐OH‐FAD (D) and NADH (E) molecules that bind with hFSP1.

**FIGURE 2:**
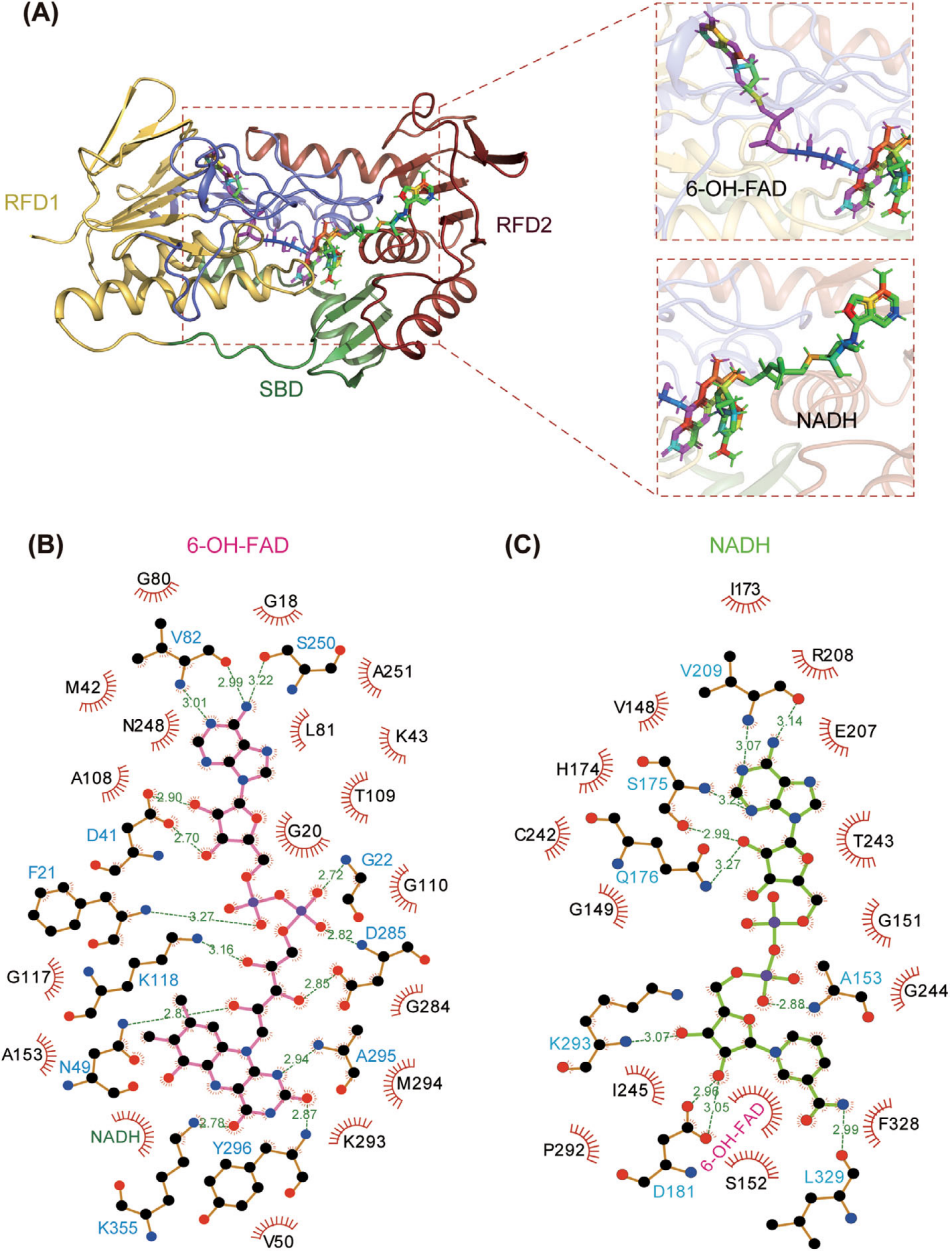
FAD‐ and NADH‐binding sites in the structure of hFSP1. (A) Overall structure of hFSP1 bound to 6‐OH‐FAD and NADH. The structure is shown as a cartoon. Yellow, Rossmann fold domains (RFD) with 6‐OH‐FAD; dark red, RFD with NADH; green, substrate binding domain (SBD). The details are shown in the red box on the right side. (B and C) Molecular interactions of 6‐OH‐FAD (B) and NADH (C) with modeled hFSP1 from ligPlot^+^. Hydrogen‐bond interactions are shown with dotted green lines, and hydrophobic interactions are displayed by red half‐moons. 6‐OH‐FAD is shown in red, and NADH is shown in green.

Utilizing the baculovirus/Sf9 cell system, we successfully expressed and purified hFSP1. The protein migrated as a single band at approximately 41 kDa on a 12% SDS–PAGE gel, which was consistent with the theoretically calculated molecular weight (Figure 1A). The elution volume of hFSP1 on gel filtration indicated that hFSP1 exists in a monomeric state in solution (Figure 1A). The purified hFSP1 protein was used for subsequent crystallization and X‐ray diffraction experiments (Figure 1B). The crystal structure of hFSP1 diffracted to a resolution of 1.75 Å (Figures 1C and S1 and Table 1). Only one molecule of hFSP1 is present in the asymmetry unit. Similar to other flavoprotein oxidoreductases,^34^, ^35^ the overall structure of hFSP1 comprised two tandem Rossmann fold domains (RFD) and a C‐terminal domain (Figures 1C and S2). The two RFDs include 6‐hydroxy‐flavin adenine dinucleotide (FAD) and nicotinamide adenine dinucleotide (reduced from, NADH), and the C‐terminal domain is responsible for substrate binding, which will be discussed later (Figures 1C and S2). In this manuscript, the C‐terminal domain is referred to as the substrate binding domain (SBD). In the structure of hFSP1, the cofactors 6‐hydroxy‐FAD and NADH were identified (Figures 1D, E and 2A). FAD is an indispensable cofactor for the oxidoreductase activity of FSP1.^27^

**TABLE 1:** Data collection and refinement statistics.

Dataset	human FSP1
Wavelength	
Resolution range	35.37–1.75 (1.813–1.75)
Space group	P 65 2 2
Unit cell	57.0372 57.0372 506.725 90 90 120
Total reflections	103,138 (9968)
Unique reflections	51,569 (4984)
Multiplicity	2.0 (2.0)
Completeness (%)	99.94 (99.72)
Mean I/sigma (I)	15.25 (3.08)
Wilson B‐factor	29.13
R‐merge	0.01441 (0.1614)
R‐means	0.02038 (0.2282)
Refinement	
Macromolecules	2788
Ligands	155
Solvent	197
Protein residues	364
RMS (bonds)	0.019
RMS (angles)	1.86
Ramachandran favored (%)	96.69
Ramachandran allowed (%)	3.31
Ramachandran outliers (%)	0.00
Rotamer outliers (%)	1.00
Clashscore	11.74
Average B‐factor	42.14
Macromolecules	42.25
Ligands	32.52
Solvent	45.47

Statistics for the highest‐resolution shell are shown in parentheses.

### 2.2 Structural analysis of hFSP1 with 6‐hydroxy‐FAD and NADH

To further determine the relationship of hFSP1 with 6‐hydroxy‐FAD and NADH, structural analysis was conducted. The interactions between 6‐hydroxy‐FAD and hFSP1 arise mainly from the contribution of hydrogen bonds and hydrophobic interactions. While adenine interacts with V82 and S250, isoalloxazine interacts with A295, Y296, and K355 (Figures 2A and B). The cyclic ribose forms hydrogen bonds with D41, and the ribitol is stabilized by N49, K118, and D285 (Figures 2A and B). The hydrogen bonds found between the two phosphate groups and F21, G22, and D285 further contribute to the interaction of 6‐hydroxy‐FAD and hFSP1 (Figures 2A and B). Hydrogen‐bond interactions also appear to be the driving force for the interaction of NADH and hFSP1. The main chains of S175 and V209 are capable of hydrogen bonding with adenine, while the main chain of L329 stabilizes nicotinamide through hydrogen bonds (Figures 2A and C). The two ribose rings of NADH are involved in hydrogen bonds with S175, Q176, D181, and K293 (Figures 2A and C), and the presence of hydrogen bonds from A153 and pyrophosphate helps the binding of NADH to hFSP1 (Figures 2A and C).

### 2.3 E156 is critical in maintaining the hFSP1 function

**FIGURE 3:**
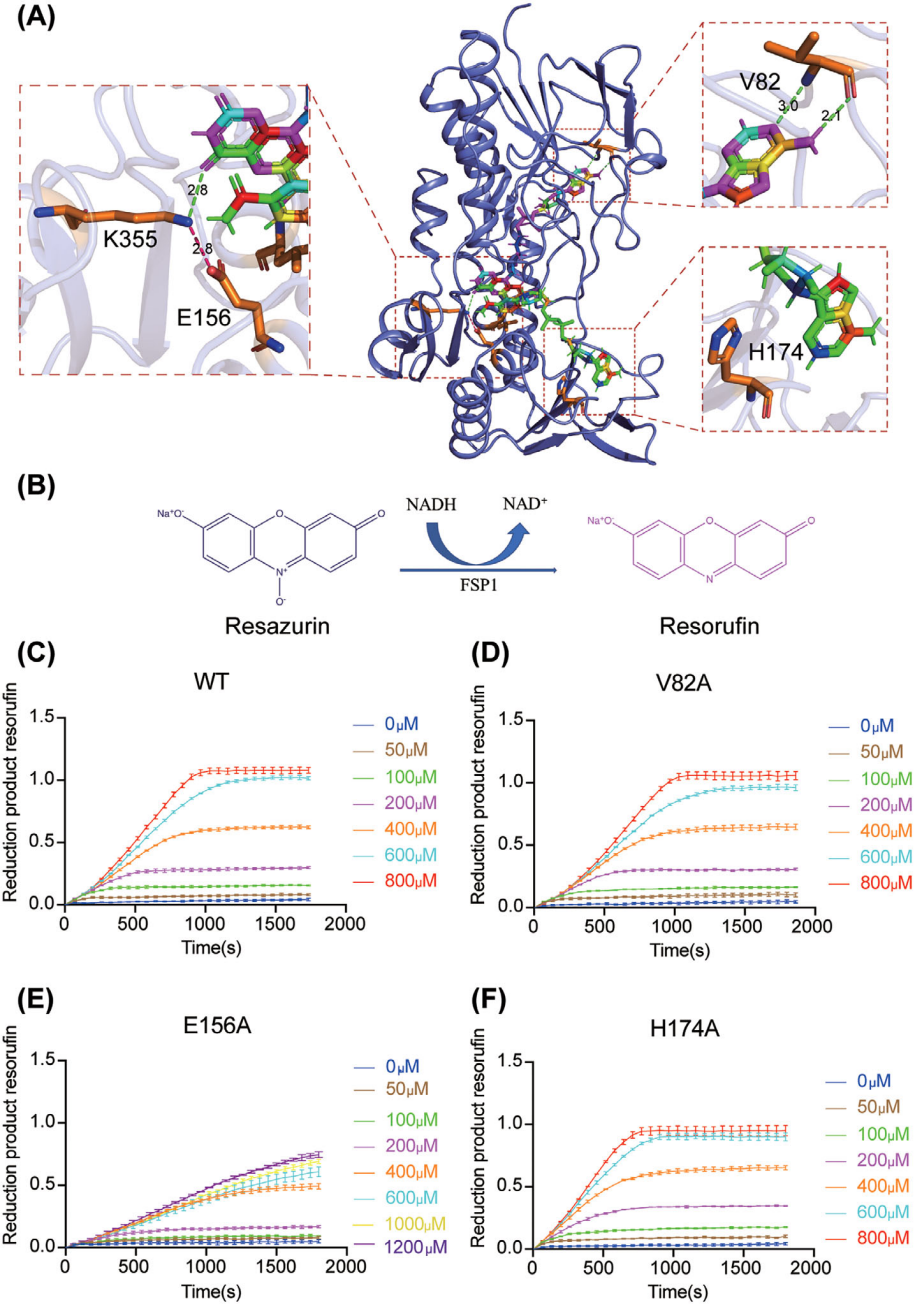
Mutational analysis of key residues responsible for hFSP1 binding to FAD and NADH. (A) Structure demonstration of key residues binding to 6‐OH‐FAD and NADH. The key residues (V82, E156, H174, and K355) on the interfaces are shown in stick models. Secondary structures are shown as cartoons. Hydrogen‐bond interactions are shown with dotted green lines and salt bridge interaction is shown with dotted red lines. (B) Schematic representation of the resazurin to resorufin conversion. Resazurin, a nonfluorescent dye colored blue, is converted to pink fluorescent resorufin in the presence of hFSP1 catalysis. (C–F) In vitro enzymatic activity measurements of hFSP1^WT^ (C), hFSP1^V82A^ (D), hFSP1^H174A^ (E), and hFSP1^E156A^ (F). In this assay, the amount of resorufin produced by hFSP1 in the presence of different concentrations of NADH was measured within 30 min. The final concentrations of hFSP1 and resazurin were 0.2 and 500 μM, respectively. The initial product of resorufin was normalized to “0” in each reaction. Data from three experiments are presented as the mean with SEM.

FSP1 is a redox enzyme, and identifying the key amino acids responsible for its functionality is critical. Therefore, to investigate key amino acids involved in hFSP1 oxidoreductase activity, three critical residues for FSP1 oxidoreductase activity were subjected to mutagenesis: V82, E156, and H174. The main chain of V82 forms a bidentate hydrogen bond with the adenine group of 6‐hydroxy‐FAD (Figures 2B and 3A). E156 is conserved and forms a salt bridge with K355, which stabilizes the hydrogen bond between the C4‐carbonyl of the isoalloxazine ring and the Nζ of K355 (Figure 3A).^27^, ^36^ H174 might contribute to the binding of NADH (Figures 2C and 3A).

Resazurin can be reduced to resorufin by hFSP1, and the amount of resorufin can be used to determine FSP1 oxidoreductase activity^26^, ^30^, ^31^, ^35^ (Figure 3B). In the fluorescence measurements, data within the linear range were selected and utilized. The initial reaction rates of wild‐type (WT) hFSP1 at different NADH concentrations were calculated (Figure 3C). The kinetic parameters were calculated using the Michaelis‒Menten equation (Table 2). As mentioned above, V82 is a potential binding site for 6‐hydroxy‐FAD. However, the *k*_cat_ value of hFSP1^V82A^ for NADH showed little difference compared with hFSP1^WT^, whereas the *km* of hFSP1^V82A^ was two‐fold higher than that of hFSP1^WT^ (Figure 3D and Table 2), indicating that the side chain of V82 has a partial effect on FSP1 activity. Notably, the *k*_cat_‐NADH of hFSP1^E156A^ significantly decreased compared with that of hFSP1^WT^, while the *K*_m_ value for NADH increased almost sixfold, indicating that E156 is critical to both the velocity and substrate preference of hFSP1 (Figure 3E and Table 2). H174 might interact with NADH through hydrophobic force. Compared with hFSP1^WT^, the *K*_m_ of hFSP1^H174A^ for NADH increased approximately fivefold, suggesting that H174 is essential to the binding of NADH/nicotinamide adenine dinucleotide phosphate (reduced form, NADPH) (Figure 3F and Table 2). FSP1 might catalyze NADH, NADPH, or both as electron donors. NADH differs from NADPH by the absence of an additional phosphate group on the 2′ position of the ribose ring that carries the adenine moiety. Intriguingly, the mutant (S175A/Q176A/R208P), losing binding with the 2′ phosphate group of NADPH, might only abolish NADPH‐mediated ferroptosis suppression.^37^ Probably, H174A might inactivate FSP1 more completely since H174A cripples the binding with the ADP group that presents in both NADPH and NADH.

**TABLE 2:** Kinetic constants of hFSP1^WT^, hFSP1^V82A^, hFSP1^E156A^, and hFSP1^H174A^ with resazurin as substrate.

hFSP1	WT	V82A	E156A	H174A
*K*_cat_ (min^−1^)	0.21	0.19	0.063	0.34
*K*_m_ (μM)	20.56	44.81	125.60	98.07
*K*_cat_/*K*_m_ (min^−1^/μM^−1^)	0.01	0.0042	0.0005	0.0035

The reaction system comprised hFSP1 (WT, 200 nM; V82A, 200 nM; H174A, 200 nM; E156A, 400 nM), resazurin (500 μM), and NADH (0‐800 μM) carried out in a reaction buffer containing 150 mM NaCl and 25 mM Tris pH 8.0. The generation of resorufin was monitored by recording the fluorescence at 585 nm, excited at 530 nm, and at 25°C.

### 2.4 Structural analysis between hFSP1 and other FSP1 protein

**FIGURE 4:**
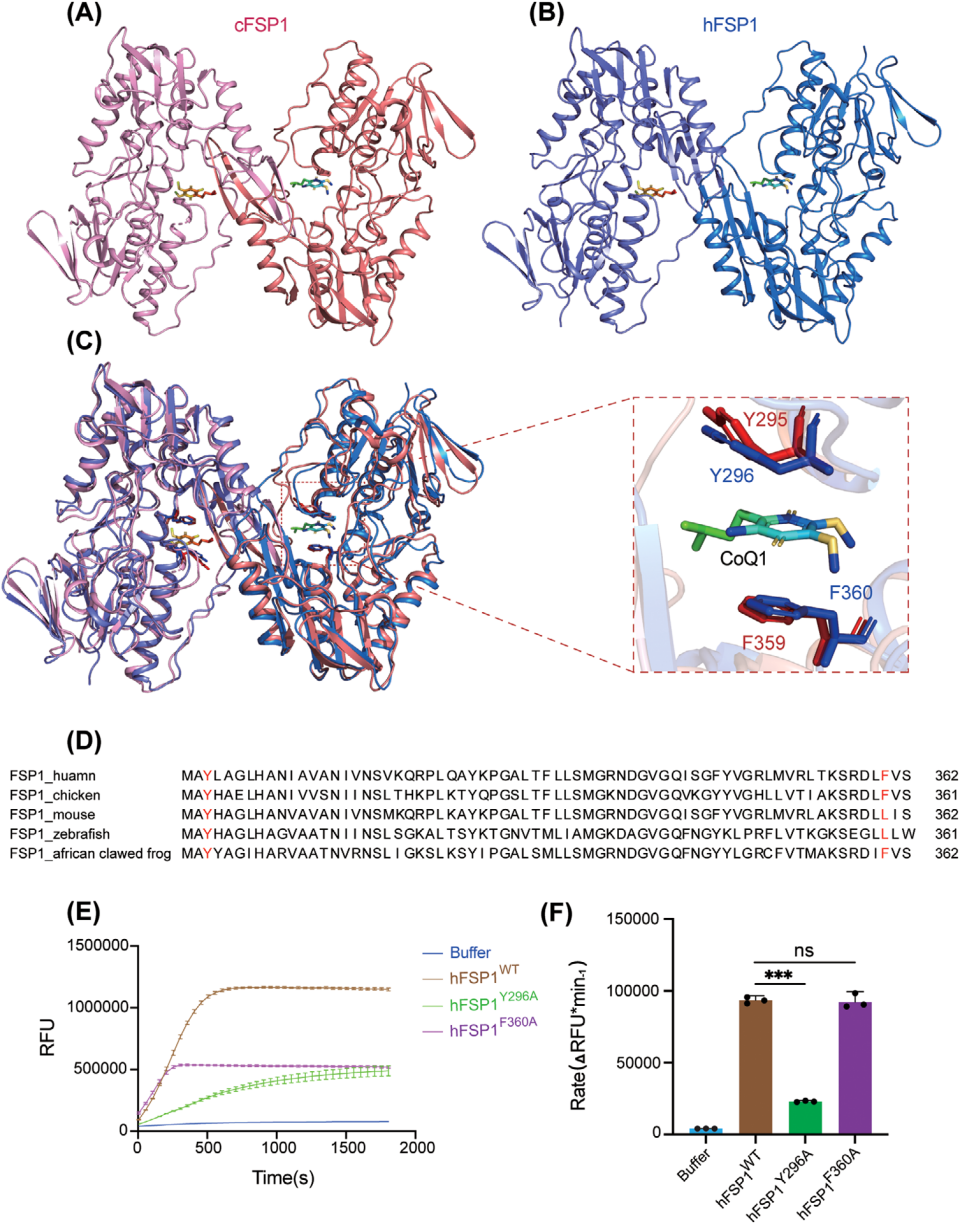
Y296 and F360 are key residues responsible for the hFSP1–CoQ_1_ interaction. (A) Cartoon representation of the overall structure of the dimeric cFSP1 in complex with CoQ_1_ (PDB: 7XPI). One protomer is colored light pink, and the other protomer is colored deep pink. A β‐barrel‐like structure formed by four β‐strands from the cFSP1 SBD mediated its dimeric assembly. (B) Cartoon representation of the overall structure of dimeric hFSP1 in complex with CoQ_1_. This model was generated based on the structural model of dimeric cFSP1 (PDB: 7XPI). One protomer is colored light blue, and the other protomer is colored deep blue. In this model, β‐barrel‐like structure‐dependent dimerization was not observed. (C) Superimposition of hFSP1 and cFSP1. The structures of hFSP1 and cFSP1 are colored in (A). The details are shown in the red box on the right side. Y296 and F360 in hFSP1 are colored blue, and corresponding Y295 and F359 in cFSP1 are colored red, which are indispensable for CoQ_1_ binding. (D) Sequence alignment of the C‐terminal domain of FSP1 in different species. Human, *Homo sapiens*; chicken, *Gallus gallus*; mouse, *Mus musculus*; zebrafish, *Danio rerio*; African clawed frog, *Xenopus laevis*. The residues responsible for binding CoQ_1_ are highlighted in red, in which Y296 is strictly conserved across species, and F360 is conserved among humans and chickens but similar across species. (E) In vitro enzymatic activity of hFSP1^WT^, hFSP1^Y296A^ and hFSP1^F360A^. In this assay, the relative fluorescence unit (RFU) of resorufin was measured within 30 min. The final concentrations of hFSP1, NADH, and resazurin were 0.2, 500, and 500 μM, respectively. (F) Statistical analysis of the in vitro enzymatic activity of hFSP1^WT^, hFSP1^Y296A^, and hFSP1^F360A^. Data from three experiments are presented as the mean with SEM. Significance was determined by one‐way ANOVA followed by Dunnett's multiple comparisons test (ns, not significant; ***, *p* < 0.001).

**FIGURE 5:**
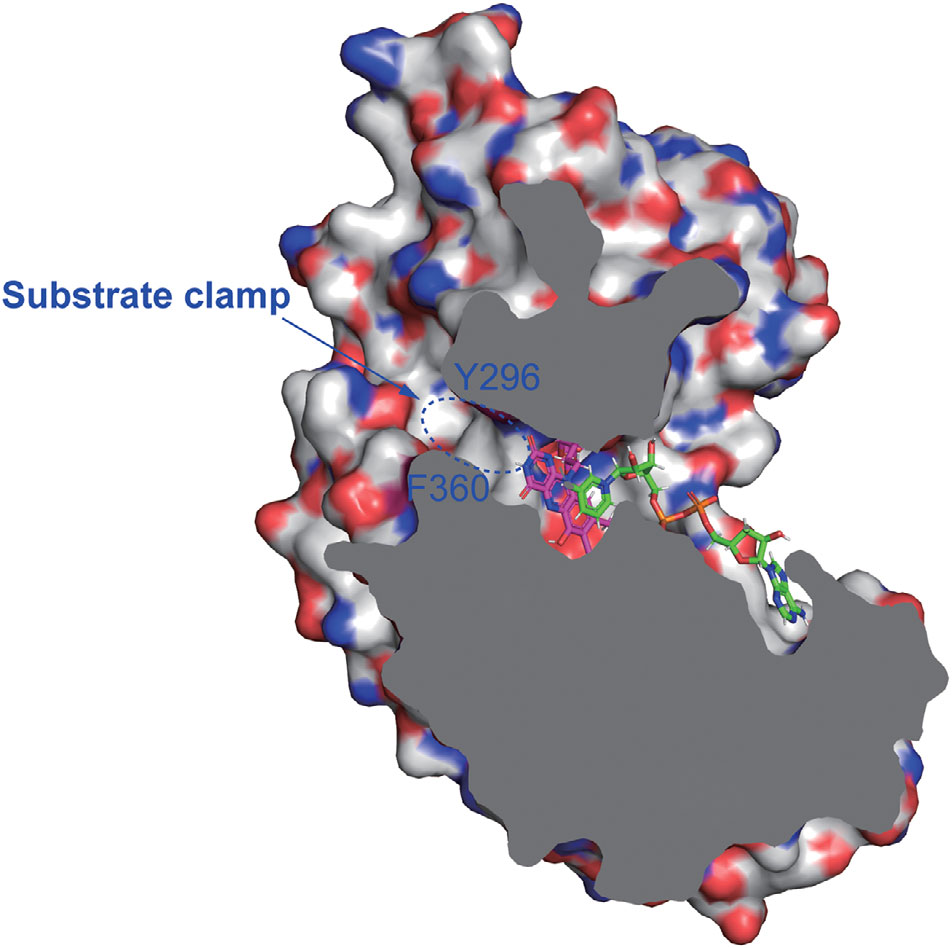
Schematic representation of the substrate clamp in hFSP1. Y296 and F360 are responsible for the formation of a substrate clamp (dotted blue circle) to mediate the interaction between hFSP1 and substrates. The overall structure of hFSP1 is shown as the electrostatic surface potential (blue positive, red negative).

While the manuscript was prepared, two FSP1 protein structures were reported.^37^, ^38^ In chicken (*Gallus gallus*) FSP1 (cFSP1), the SBD of cFSP1 features two protruding β‐strands (β17–β18) extending into a cavity of the other monomer, possibly contributing to the formation of an active pocket.^39^ Additionally, the cFSP1 SBD displayed a characteristic arrangement of four β‐strands forming a β‐barrel‐like structure, facilitating interactions between the two cFSP1 monomers, ultimately resulting in dimerization (Figure 4A). The authors suggested that dimerization is likely a prerequisite for proper FSP1 function.^39^ However, in hFSP1, such β‐strands are absent within the SBD, and our gel filtration chromatography results suggested that hFSP1 is monomeric in solution (Figures 1A and 4B). Model analysis showed that Y296 and F350 were critical in the formation of the binding pocket for the hFSP1 substrate quinone (Figure 4C). Sequence alignment of the SBD of FSP1 (295–362) revealed that these two amino acids are conserved across species (Figure 4D). Furthermore, the hFSP1 mutants Y296A and F360A showed impaired oxidoreductase activity, indicating that the SBD of hFSP1 responds to substrate binding (Figures 4E and F). Hence, removing the SBD of hFSP1 might disrupt the binding of substrate, which thereby cripples the activity of FSP1. In summary, the SBD is critical to substrate binding. Considering that FSP1 is highly conserved from fish to mammals, it is rational to name the C‐terminal domain the SBD (Figure 5).

## 3 DISCUSSION

While conventional anticancer therapies aim to induce ferroptosis by inhibiting GPX4, in vivo results have revealed inadequate pharmacokinetic responses and unsatisfactory effects. Notably, GPX4 deficiency is embryonically lethal in mice, making the targeting of FSP1 a potentially safer choice in cancer treatment.^40^ In Kelch‐like ECH‐associated protein 1 (KEAP1)‐mutant lung cancer, as well as other cancer cells with mutations affecting Kristen Rat Sarcoma Viral oncogene homolog (KRAS), researchers have observed significant upregulation of FSP1 as a protective response through nuclear factor erythroid 2‐related factor 2 (NRF2).^33^, ^41^ Yuan et al.^42^ identified YTH N6‐methyladenosine RNA binding protein C1 (YTHDC1) as a tumor progressive suppressor through the modulation of FSP1 mRNA stability. Additionally, Gong et al.^43^ found that tripartite motif containing 21 (TRIM21) binds to FSP1 and mediates its ubiquitination on K322 and K366 residues through K63 linkage in gastrointestinal tumors, leading to tumor progression. However, despite the critical role of FSP1, limited FSP1 inhibitors have been identified so far, with iFSP1 being the most popular one.^24^ The molecular mechanisms underlying its effect, as well as its target sites within FSP1 essential for ferroptosis inhibition, remain to be explored. This is primarily attributed to the unresolved crystal structure of FSP1. Elucidating the structure of hFSP1 would offer valuable insights into the functional mechanism and provide an accurate model for drug design and development.

In this study, we reported the crystal structure of hFSP1 containing FAD and NADH at a resolution of 1.75 Å, which is the highest resolution structure of FSP1 to date and will benefit drug development targeting FSP1. FSP1 can use NADH or/and NADPH as electron donor substrates and purified FSP1 proteins showed no obvious preference for NADH or NADPH oxidation.^39^ As NADH and NADPH are structurally similar except for an additional phosphate group in NADPH, the structural basis of interaction between hFSP1 and NADH revealed that H174A might abolish FSP1 activity more completely. Despite the subcellular location of NADH and NADPH might be different in specific cells, the NADH locates and functions mainly in the mitochondria, while the NADPH locates in the cytosol.^44^ Our results might provide additional information in studying FSP1 in mitochondria.

Additionally, we measured the catalytic constants of hFSP1 and evaluated the effect of residues involved in the oxidoreductase activity of hFSP1. The results indicated that some residues, for example, the side chain of V82, showed a partial effect on the activity of hFSP1. Recently, two papers from Conrad's laboratory, published in *Nature* and *Nature Structural & Molecular Biology*, have also shed light on the structural mechanism underlying FSP1 oxidoreductase activity.^45^, ^46^ Nakamura et al. conducted a comprehensive study on various untargeted FSP1 random mutations in cancer cells, pinpointing the essential role of G244 in the predicted NAD(P)H‐binding site.^46^ They also identified K355 and E160 as crucial for FSP1's proton‐transfer function. Notably, their investigation revealed that iFSP1 specifically binds to phenylalanine at position 360 in the quinone‐binding pocket of human, chicken, and frog FSP1. Furthermore, they discovered a new species‐independent inhibitor, viFSP1, which binds to the NADPH‐binding pocket. Mutations of amino acids within this pocket (A153, F328, M294, and T327) resulted in reduced FSP1 activity.^45^ In a separate study, Mishima et al.^45^ explored that DHODH inhibitors, particularly the high concentration of Brequinar, could sensitize cancer cells to ferroptosis by inhibiting FSP1. This information provides a crucial mechanistic rationale for understanding and maintaining FSP1 function.

Consistent with the results of gel filtration, hFSP1 exhibited a monomeric state in the asymmetric unit of the crystal instead of forming a dimer as cFSP1.^37^ This difference is likely attributed to hFSP1 lacking two β‐strands of the SBD in comparison with cFSP1. Compared with AIFM1, which has been identified as a dimer in its reduced state, hFSP1 does not show sequence and structure conservation in those elements (including the C‐loop, E246, and R449) that are responsible for AIFM1 dimerization.^47^ Our data support that hFSP1 functions as a monomer, aligning with a recent report.^38^ Importantly, based on the modeling, we identified two conserved residues, Y296 and F360, as crucial for substrate binding. Y296 and F360 together clamp the quinone group of CoQ, and mutagenesis of Y296 and F360 disrupted the activity of hFSP1. Since both residues belong to the C‐terminal domain, we suggest naming the C‐terminal domain of hFSP1 the SBD. Cautiously, deleting the hFSP1 CTD also leads to the removal of Y296 and F360, thereby impairing substrate binding and disrupting the oxidoreductase activity of hFSP1.

The oligomeric statuses of flavoprotein oxidoreductases are diverse and might change according to subcellular localization.^34^, ^35^ Although we purified the full‐length protein of hFSP1, the N‐terminus is disordered and invisible in the structure; however, the myristoylation of the hFSP1 N‐terminus mediates the localization of hFSP1 on the plasma membrane and lipid droplets.^27^ The oligomeric status of hFSP1 might change once it tethers on the membrane. Nevertheless, in the solution, the data in this manuscript together with a previous study suggest that hFSP1 functions as a monomer in solution.^38^

Our research sheds light on the molecular mechanism of how hFSP1 recognizes and binds CoQ. In the future, more effort should be devoted to identifying potent hFSP1 activators or inhibitors based on the findings here and validating their biological effects in vitro and in vivo. This research could support novel therapeutics for the clinical treatment of diseases associated with ferroptosis.

## 4 MATERIALS AND METHODS

### 4.1 Expression and purification of the hFSP1 protein

We used the baculovirus/Sf9 system to express hFSP1 (1−373) as previously described.^48^ The DNAs of WT and mutated hFSP1 were subcloned and inserted into the pFastBac Dual vector under the control of the polyhedron promoter with an N‐terminal His_8_‐MBP‐tag that can be removed by Tobacco Etch Virus (TEV) protease. Baculoviruses were generated in Sf9 cells with the bac‐to‐bac system (Life Technologies) following the manufacturer's instructions. The cells were infected and harvested after 60 h. Cells were pelleted by centrifugation at 2000×*g* for 15 min. The pellets were lysed in 25 mM Tris–HCl pH 8.0, 150 mM NaCl, 0.5 mM TCEP–HCl, and 1 mM PMSF by sonication. The lysate was then centrifuged at 25,000 × g for 30 min at 4°C. The supernatant of hFSP1 was loaded onto Ni‐NTA resin at 4°C and eluted with 25 mM Tris–HCl pH 8.0, 150 mM NaCl, 0.5 mM TCEP–HCl, and 250 mM imidazole pH 8.0. The eluate of target proteins was digested with TEV protease at 4°C overnight after dialysis to remove imidazole. TEV and His_8_‐MBP were removed by applying the solution to a Hi‐Trap Q HP column. Peak fractions were pooled and further purified on a Superdex 200 10/300 GL column equilibrated with 25 mM Tris–HCl pH 8.0, 150 mM NaCl, and 2 mM DTT, after which the target proteins were pooled and flash‐frozen in liquid N2 for storage.

### 4.2 Crystallization of hFSP1

Purified hFSP1 (Figure 1A) was concentrated to 8 mg/mL with a 10‐kDa cutoff centrifugal filter (Millipore). The crystals of the hFSP1 protein were obtained through hanging‐drop vapor diffusion at 18°C. The protein solution was mixed with a well buffer containing 0.1 M imidazole (pH 8.0) and 1 M sodium citrate. Crystals emerged within 24 h and reached their full size within 2−3 days. These crystals were flash‐frozen in well buffer supplemented with 20% glycerol and were used for subsequent data collection.

### 4.3 Data collection and structure determination

Diffraction data were collected on BL18U and BL19U at the Shanghai Synchrotron Radiation Facility (SSRF) and subsequently processed using HKL2000.^49^ The structure was solved using molecular replacement using the Alphafold prediction model (AF‐Q9BRQ8‐F1) as the search model.^50^, ^51^ Molecular replacement was carried out with the program PHENIX.^52^ Model building and refinement were carried out with COOT and PHENIX.^52^, ^53^ All structural representations were generated using PyMOL software.^54^ The final FSP1 structure was determined at a resolution of 1.75 Å and deposited in the Protein Data Bank (PDB) with the accession entry 8WIK. Comprehensive statistics for data collection, structure determination, and refinement are provided in Table 1.

### 4.4 FSP1 enzyme activity assay

The FSP1 enzyme activity was carried out as previously reported.^25^ Simply, the reaction system comprises 50 μL hFSP1 (WT, 200 nM; V82A, 200 nM; H174A, 200 nM; E156A, 400 nM), 25 μL resazurin (500 μM), and 25 μL NADH (0–800 μM). The components were diluted in a reaction buffer that contained 150 mM NaCl and 25 mM Tris pH 8.0. The generation of resorufin was monitored by recording the fluorescence at 585 nm, excited at 530 nm, and at 25°C over a period of 1800 s, with data recorded at 1‐min intervals. Data collection was conducted using a 96‐well plate (Corning, 3701) in a BioTek Synergy2 instrument. Subsequent data analysis was performed employing GraphPad software.

## AUTHOR CONTRIBUTIONS

S. Q. and B. L. provided the research concepts and designed the experiments, revised, and edited the manuscript; S. F., X. H., and D. T. conducted the experiments, analyzed the data, and drafted the manuscript; X. L. performed parts of experiments; L. O., D. Y., and K. W. revised and edited the manuscript. All authors have read and approved the final manuscript.

## CONFLICT OF INTEREST STATEMENT

The authors declare that they have no conflict of interest.

## ETHICS STATEMENT

Not applicable.

## Supporting information

Supporting Information

## Data Availability

The FSP1 structure was deposited in the PDB with the accession entry: 8WIK. Raw data could be obtained upon request from corresponding authors (Shiqian Qi, qishiqian@scu.edu.cn and Banghua Liao, liaobanghua@wchscu.cn).
