# Supplementary material for: The crystal structure of human ferroptosis suppressive protein 1 in complex with flavin adenine dinucleotide and nicotinamide adenine nucleotide
Source: MedComm (2020). 2024 Feb 26;5(3):e479. doi: 10.1002/mco2.479 (PMC10896247; doi:10.1002/mco2.479)
Supplement: Supplementary file 1 — Supporting Information [file MCO2-5-e479-s001.docx]

**The Crystal Structure of Human FSP1 in Complex with FAD and NADH**

Supplementary information

Shijian Feng^1, #^, Xiaofang Huang^1, #^, Dan Tang^1, #^, Xiaoyu Liu^1^, Liang Ouyang^1^, Dehua Yang^2^, Kunjie Wang^1^, Banghua Liao^1, *^, Shiqian Qi^1, *^

1. Department of Urology and Institute of Urology (Laboratory of Reconstructive Urology), State Key Laboratory of Biotherapy and Cancer Center, West China Hospital, College of Life Sciences, Sichuan University, Chengdu, China.
2. The National Center for Drug Screening, Shanghai Institute of Materia Medica, Chinese Academy of Sciences, Shanghai, China

Corresponding Author: Shiqian Qi, qishiqian@scu.edu.cn, Banghua Liao, liaobanghua@wchscu.cn

**
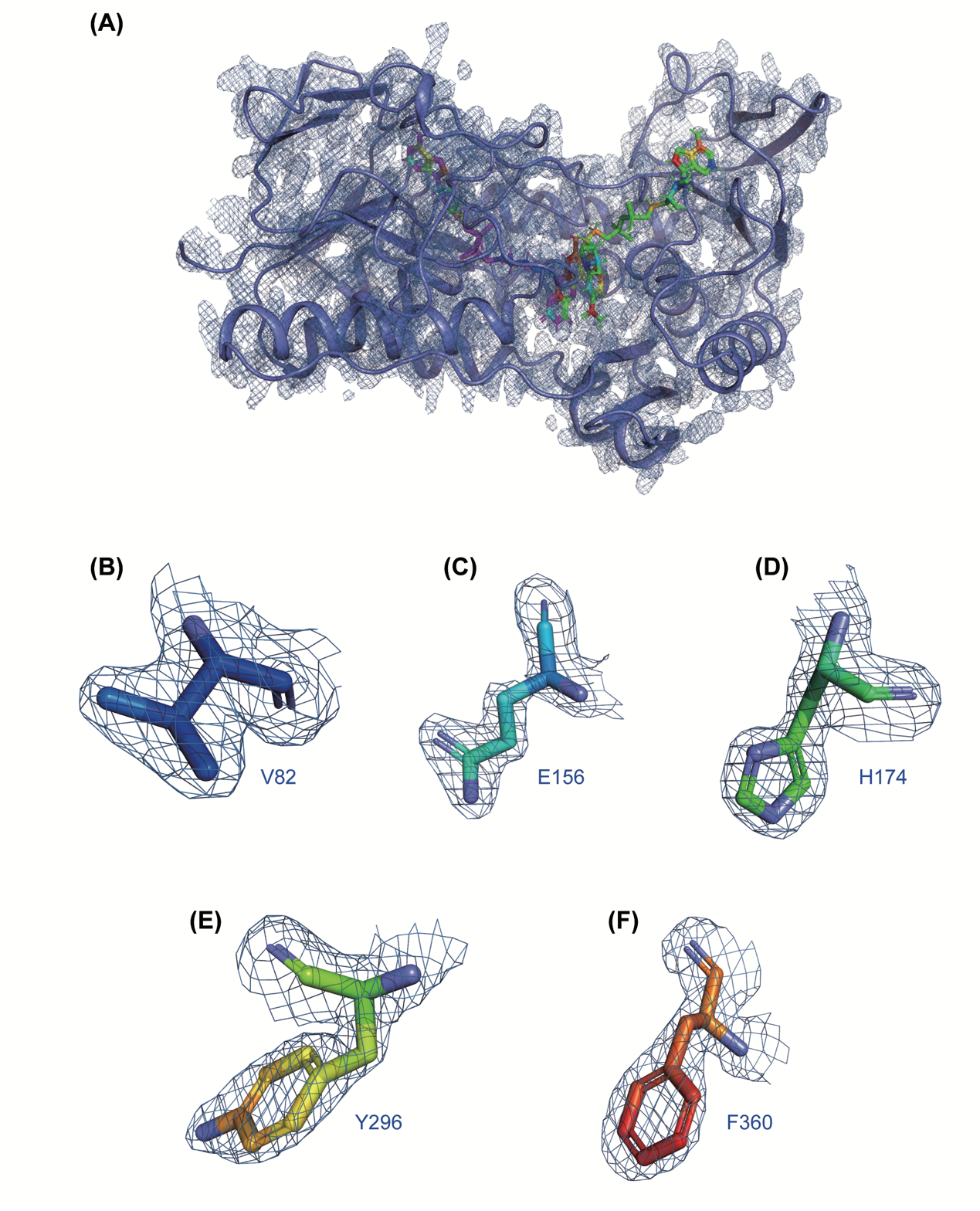
**

Figure S1. Electron density maps of hFSP1.

(A-F) The model of hFSP1 and key amino acid residues fit well with the electron density. 2FO-FC electron density maps contoured at the 1.5 σ level around hFSP1 and the indicated regions.


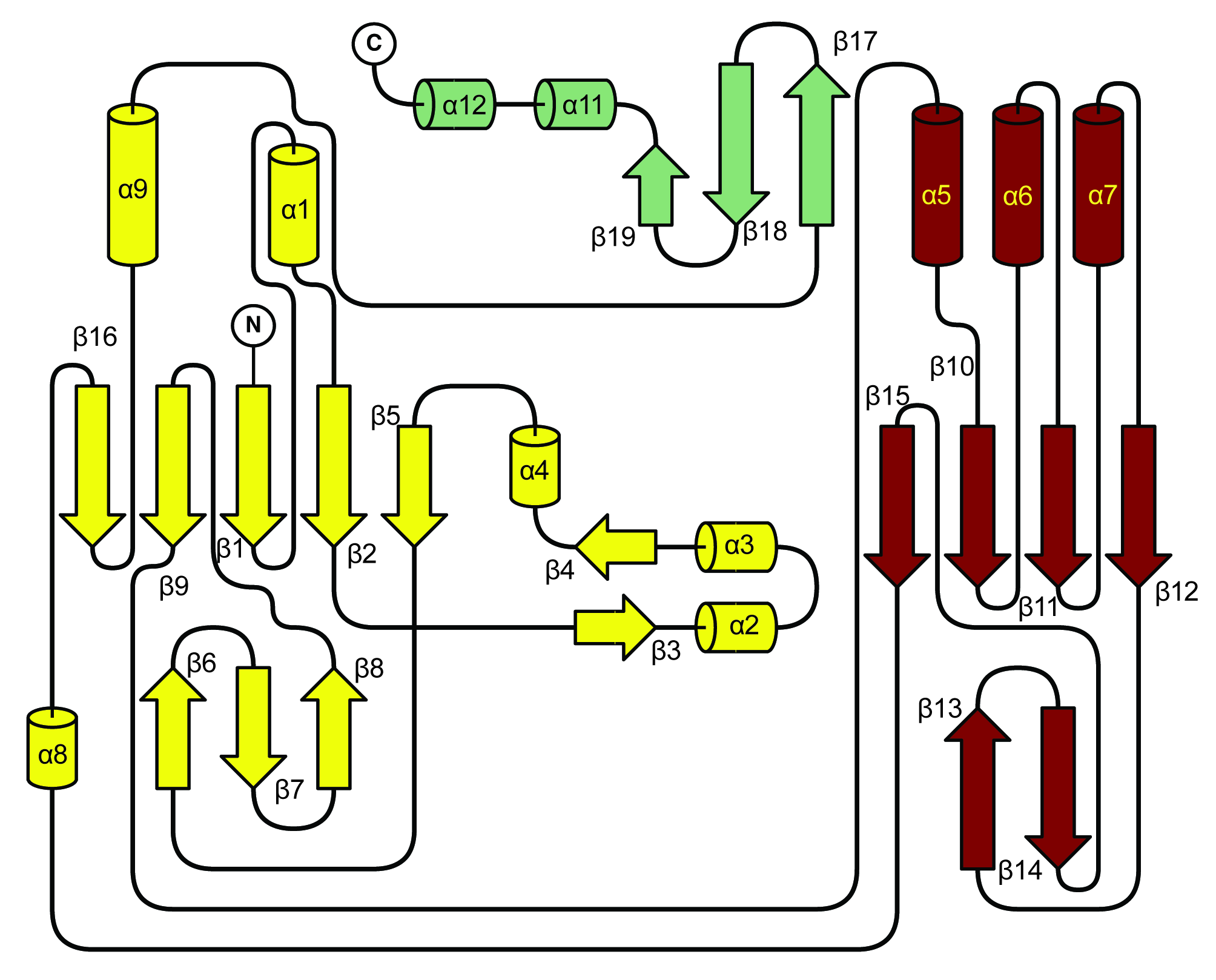


Figure S2. The topology diagram of the hFSP1 structure.

β strands are indicated as arrows, and α helices are indicated as cylinders. β strands and α helices in the first (N-terminal) RFD, the second RFD, and the SBD are colored yellow, red, and green, respectively. All other loop regions are colored black. The figure was generated using TopDraw, a program within the CCP4 suite^55^.
